# Residue Mutations in Murine Herpesvirus 68 Immunomodulatory Protein M3 Reveal Specific Modulation of Chemokine Binding

**DOI:** 10.3389/fcimb.2019.00210

**Published:** 2019-06-25

**Authors:** Radka Šebová, Vladena Bauerová-Hlinková, Konrad Beck, Ivana Nemčovičová, Jacob Bauer, Marcela Kúdelová

**Affiliations:** ^1^Department of Viral Immunology, Biomedical Research Center, Institute of Virology, Slovak Academy of Sciences, Bratislava, Slovakia; ^2^Department of Biochemistry and Structural Biology, Institute of Molecular Biology, Slovak Academy of Sciences, Bratislava, Slovakia; ^3^Cardiff University School of Dentistry, Heath Park, Cardiff, United Kingdom

**Keywords:** MHV-68, M3 protein, site-directed mutagenesis, chemokine binding, CCL5, CXCL8, complex modeling, molecular recognition

## Abstract

The M3 protein (M3) encoded by murine gammaherpesvirus 68 (MHV-68) is a unique viral immunomodulator with a high-affinity for a broad spectrum of chemokines, key mediators responsible for the migration of immune cells to sites of inflammation. M3 is currently being studied as a very attractive and desirable tool for blocking the chemokine signaling involved in some inflammatory diseases and cancers. In this study, we elucidated the role of M3 residues E70 and T272 in binding to chemokines by examining the effects of the E70A and T272G mutations on the ability of recombinant M3, prepared in *Escherichia coli* cells, to bind the human chemokines CCL5 and CXCL8. We found that the E70A mutation enhanced binding of M3 to CCL5 two-fold but had little effect on its binding to CXCL8. In contrast, the T272G mutation was found to be important for the thermal stability of M3 and significantly decreased M3's binding to both CCL5 (by about 4×) and CXCL8 (by about 5×). We also constructed *in silico* models of the wild-type M3–CCL5 and M3–CCL8 complexes and found substantial differences in their physical and chemical properties. M3 models with single mutation E70A and T272G suggested the role of E70 and T272 in binding M3 protein to chemokines. In sum, we have confirmed that site-directed mutagenesis could be an effective tool for modulating the blockade of particular chemokines by M3, as desired in therapeutic treatments for severe inflammatory illnesses arising from chemokine network dysregulation.

## Introduction

Chemokines are low molecular weight, chemoattractant cytokines (classified into four subfamilies—CC, CXC, CX_3_C, and C according to the position of their N-terminal cysteine residues) that modulate the migration of immune cells from blood vessels to sites of infection and inflammation, an important phenomenon in host defense (Zlotnik and Yoshie, 2000). Chemokines establish gradients through specific interactions with glycosaminoglycans (GAGs) expressed on endothelial cell surfaces (Proudfoot et al., 2003) and direct target cell migration and activation by binding to G-protein-coupled chemokine receptors (GPCRs) (Sallusto and Baggiolini, 2008; Burg et al., 2015). Thus, chemokines play an important role in host defense against invading pathogens. It has been established that chemokines are associated with disease progression and are also implicated in hematopoiesis, angiogenesis, and development. Besides their well-documented role in many inflammatory diseases, chemokines also have a role in cancer development (Chow and Luster, 2014).

Given the central role that chemokines play in antiviral defense, it is not surprising that many viruses have evolved strategies to alter host chemokine function to their benefit (Alcami and Koszionowski, 2000). Viruses have acquired and optimized molecules that interact with the host chemokine network. They express a repertoire of proteins which interfere with the host immune response in order to avoid being eliminated from the organism. Virus-encoded immunomodulatory proteins target or bind to chemokines and their receptors and thus specifically modulate chemokine gradient formation and ligand-receptor recognition; they even have the potential to completely block chemokine-mediated responses to viral infection. They are used to promote cell entry, facilitate dissemination of infected cells, and interfere with extracellular chemokines (Alcami, 2003).

Virus-encoded immunomodulatory proteins have been identified from many virus families, with the majority being derived from DNA viruses, from which poxviruses and herpesviruses have evolved to encode the largest number of immunomodulators, called viral chemokine binding proteins (*v*CKBPs) (Alcami and Lira, 2010). So far, five *v*CKBP subfamilies have been described, which differ in specificity as well as in chemokine interaction mechanisms. However, only a few of them have been tested for their therapeutic potential *in vivo* even though neutralizing chemokine signaling is a very attractive therapeutic strategy for many diseases. The most recently discovered class of herpesvirus immunomodulators include the only two known chemokine-binding proteins encoded by gammaherpesviruses. They are the M3 protein from murine gammaherpesvirus 68 (MHV-68) and the R17 protein from the rodent herpesvirus Peru, which both show no significant homology to mammalian proteins (Heidarieh et al., 2015; González-Motoz et al., 2016).

MHV-68, from the genus *Rhadinovirus* (Van Regenmortel et al., 2000), closely related to human gammaherpesviruses (Kúdelová and Rajčáni, 2010), was isolated from murid rodents of *Myodes* spp. captured in the former Czechoslovakia (Blaškovič et al., 1980). In addition to MHV-68, the closely related MHV-72, and MHV-4556 strains have also been thoroughly studied with respect to their pathogenicity and molecular properties (Rajčáni and Kúdelová, 2007). Recently, MHV-68 pathogenesis was also shown in ticks, thereby making MHV-68 an arthropod-borne virus (arbovirus) (Hajnická et al., 2017; Kúdelová and Štibrániová, 2019). Most recent studies, however, have focused on the immunomodulatory M3 (44 kDa) encoded by MHV-68, which has an exceptional ability among viral immunomodulators to bind a wide range of chemokines (van Berkel et al., 2000).

The M3 protein was found to be the first example of a soluble inhibitor encoded by a herpesvirus (secreted from cells in large amounts during MHV-68 infection) and is currently the only such protein known to bind and inactivate chemokines from all four chemokine subfamilies. It specifically interacts with the N-terminal chemokine binding domain of the GPCR, thereby blocking receptor recognition and inhibiting chemokine-mediated leukocyte migration (Alexander et al., 2002; Sarawar et al., 2002; Webb et al., 2003). So far, M3 was shown *in vivo* to reduce mononuclear cellular responses after MHV-68-induced meningitis in mice (van Berkel et al., 2002). Along with studies exploring its molecular properties, a variety of animal models have been developed to test the biological and pharmaceutical properties of M3 (Lira et al., 2009), but they mainly relate to its potential use in gene therapy. Jensen et al. (2003) demonstrated that M3 expression in the pancreas of mice inhibits recruitment of lymphocytes induced by transgenic expression of CCL21 in this organ. Induction of M3 gene expression resulted in a 67% reduction in intimal area, suggesting that M3 may be effective in attenuating the intimal hyperplasia associated with arterial stenosis (Pyo et al., 2004). M3 expression overcame the cellular inflammatory responses in rat hepatocellular carcinoma lesions induced by a recombinant oncolytic vesicular stomatitis virus, prolonged the therapeutic effect of this virus, and improved animal survival (Wu et al., 2008). Recombinant M3 also inhibited angiogenesis and neovascularization (Andrés et al., 2009). Studies on double transgenic mice expressing both M3 and different chemokines in pancreatic islets explored the role of chemokines and the effects of M3 on the development of *diabetes mellitus* type I. They showed that M3 can effectively inhibit mixed populations of chemokines preventing diabetes development in mice (Martin et al., 2007). Millward et al. (2010), using an experimental autoimmune encephalomyelitis animal model, showed that M3 expressed directly in the CNS significantly reduced the number of immune cells infiltrating the CNS as well as the clinical severity of the disease, suggesting M3 could be a novel therapeutic for neuroinflammatory diseases. M3 has therefore been suggested as a novel tool for blocking chemokine signaling, which is highly desirable for anti-inflammatory therapies.

The earliest studies on the biological properties of the 406-residue MHV-68 M3 showed that it bound to a broad spectrum of chemokines, with the lowest binding affinities to members of the CXC subfamily (van Berkel et al., 2000). Our previous studies on the MHV-68 M3 and MHV-72 M3, either secreted into the media of infected BHK-21 cells or prepared recombinantly in *E. coli* cells, showed that M3 binds very strongly to only one of the human chemokines tested, CCL5 (other tested ones included CCL2, CCL3, CCL5, CCL11, and CXCL8) (Belvonciková et al., 2008; Matúšková et al., 2015). Studying the crystal structure of the M3–CCL2 complex (also known as monocyte chemoattractant protein 1 or MCP-1) helped to describe how this viral protein might be able to bind chemokines despite having no amino-acid sequence homology to host chemokine receptors (Alexander et al., 2002). Due to the complex structure of M3 and the broad spectrum of chemokines it can potentially bind, the exact functions of its individual domains remain poorly understood.

Early studies on MHV-72 revealed a single residue substitution, D307G, in M3, which lies close to the C-terminal M3–CCL2 binding region (Belvonciková et al., 2008). Unlike MHV-68, MHV-72 elicits a weaker immune response but shows stronger oncogenicity in experimentally infected mice. Additional studies on MHV-72 M3 purified from the media of infected BHK-21 cells showed that its binding to CCL5 and CXCL8 was about 10 and five times lower, respectively, than the binding of MHV-68 M3. Recent biochemical studies on recombinant M3's prepared in *E. coli* cells showed that the D307G substitution in MHV-72 M3 was responsible for a large decrease in its CCL3 binding relative to MHV-68 M3 (Matúšková et al., 2015). Our more recent study of recombinant MHV-68 M3 prepared from insect cells revealed that the deletion of the M3 signal peptide allows stronger binding to CCL5, but not to CXCL8 (Šebová et al., 2017). Taken together, our results prompted this work to investigate the impact of particular amino-acid substitutions which are not part of the M3–chemokine interface on the ability of MHV-68 M3 to bind to individual chemokines using site-directed mutagenesis.

The aim of this study was to better clarify the structural basis for the specific activities of M3 by identifying those residues which could alter its binding to individual chemokines, and those mutations which either enhance or suppress these activities. We selected and introduced two unique mutations into MHV-68 M3, one located in its N-terminal domain (NTD)—E70A—and another in its C-terminal domain (CTD)—T272G. To determine the effect of these mutations on chemokine binding, we prepared two mutant recombinant M3's incorporating each mutation individually and investigated their effects on the binding of the CCL5 and CXCL8 chemokines. We examined the effect of the most effective mutation, T272G, on the thermal stability and folding of M3 by CD spectroscopy. To understand the effect of both mutations on chemokine binding, we created and analyzed *in silico* models of the M3 E70A and T272G mutants. To gain better insight into M3 chemokine recognition, we created new *in silico* models of complexes wtM3 with CCL5 and CXCL8 and compared them to the known X-ray crystal structures of the wtM3–CCL2 and wtM3–XCL1 complexes.

## Materials and Methods

### Virus and Cells

MHV-68 originally isolated from *Myodes glareolus* was kindly provided by Prof. Mistríková (Comenius University, Slovakia). MHV-68 was subsequently twice plaque-purified to obtain clone f2.6 and then propagated using BHK-21 fibroblasts (ATCC number: CCL-10) as described previously (Raslova et al., 2001). Cell cultures were maintained in Dulbecco's Minimum Essential Medium (Gibco) supplemented with 10% (v/v) fetal bovine serum (FBS), 2 mmol/l glutamine (Invitrogen) and penicillin–streptomycin–amphothericin (Cambrex) at 37°C. *E. coli* Rosetta-gami 2 (DE3) competent cells (Novagen) were used to express recombinant M3's following the manufacturer's instructions.

### Cloning and Site-Directed Mutagenesis

Pure MHV-68 virion DNA, purified as previously described (Raslova et al., 2001) was used as a template to amplify a fragment encoding the full-length MHV-68 M3 protein sequence carrying a His-tag anchor at its C-terminal end as described in Pančík et al. (2013). This DNA fragment was cloned into a pET26b(+) expression vector (Novagen) to create the recombinant plasmid P26-M3his/68. Next, two expression vectors with different single mutations to the M3 gene (P26-M3his/68E70A and P26-M3his/68T272G) were prepared using primers incorporating mutations (Table S1), together with the Stratagene QuickChange site-directed mutagenesis kit (Agilent Technologies) according to the manufacturer's instructions. Both strands of all expression plasmids used here were sequenced to confirm the cloned fragments had the correct sequences (BITCET, Slovakia).

### Expression, Purification and Immunoblotting of wtM3, M3-E70A, and M3-T272G Proteins

To produce each recombinant M3, we followed the optimized protocol previously described (Matúšková et al., 2015) with slight differences. Briefly, the expression of recombinant M3 from *E. coli* competent cells was induced by IPTG at a final concentration of 0.5 mmol/l and then carried out for 3 h at 37°C. The cells were sonicated in lysis buffer containing 50 mmol/l Tris-HCl pH 8, 500 mmol/l NaCl, 10% (w/v) glycerol, 10 mmol/l imidazole, and 0.2% (w/v) sulfobetaine-14 (Sigma-Aldrich). Soluble His-tagged recombinant protein was then purified from the lysate supernatant using IMAC with HIS-Select Cobalt Affinity Gel (Sigma Aldrich). To elute M3, buffer A containing 50 mmol/l Tris-HCl pH 8, 500 mmol/l NaCl, 10% (w/v) glycerol, 250 mmol/l imidazole, and 0.2% (w/v) sulfobetaine-14 was used. The amount of M3 in the elution fractions was quantified using a NanoDrop 2000 spectrophotometer (Thermo Fisher Scientific). SDS-PAGE and western blotting were performed to confirm the size, purity and specificity of M3 recovered using the anti-M3 antibody 1/27 (Šebová et al., 2017).

### ELISA

Recombinant proteins wtM3, M3-E70A, and M3-T272G were tested for their ability to inhibit commercial recombinant CCL5 and CXCL8 using the Human CCL5/RANTES and Human CXCL8/IL-8 DuoSet ELISA kits (R&D Systems). Each M3–chemokine interaction was tested in three independent experiments. In ELISA assays, 50 pg of a given recombinant chemokine was mixed with six different amount of tested recombinant M3 (2, 10, 20, 100, 200, and 400 ng) to achieve final concentrations: 4.5 × 10^−11^, 2.25 × 10^−10^, 4.5 × 10^−10^, 2.25 × 10^−9^, 4.5 × 10^−9^, and 9 × 10^−9^ mol/l in a volume of 110 μl. Each mixture was incubated at 21°C for 1.5 h with gentle shaking and then applied to ELISA plates (Greiner Bio-one) following the manufacturer's instructions, but using *ortho*-phenylenediamine as the substrate. Each sample (examined in triplicate) was measured using an ELISA reader (EL × 808, BioTek) with buffer A serving as a negative control. A reduction in the sample's OD_492_ relative to that of 50 pg chemokine without M3 was determined as the rate of chemokine inhibition (%) by M3 present in assay in given concentration. To compare the strength of binding of wtM3, M3-E70A, and M3-T272G to chemokine, data were expressed as IC_25_ or IC_50_ interpreting the quantity of the given protein needed to remove 25 or 50% of a given chemokine from solution, in other words protein concentration that inhibit 25 or 50% of chemokine used in the assay. Statistical significance was determined using a two-tailed unpaired Student *t*-test ^***^, *p* < 0.001 (GraphPad Software), using as null that there was no difference in binding relative to wtM3.

### Circular Dichroism Spectroscopy

CD spectra were recorded on an Aviv Model 215 spectrometer (Aviv Biomedical Inc., Lakewood) equipped with a thermostatted cell holder. Proteins were dialyzed into a buffer containing 30 mmol/l Tris-SO_4_, pH 8.0, 250 mmol/l NaF, 0.1% sulfobetaine 14 (w/v), and 3% glycerol (v/v) at 4°C. Far-UV spectra (260–185 nm) were collected in a 0.2 (wtM3) or 0.5 mm (M3–T272G) quartz cuvette at 4, 16, 25, 35, 45, 50, 60, 70, and 80°C (80°C for wtM3 only) at protein concentrations of 0.35 (wtM3) or 0.17 mg/ml (M3–T272G), with 4 s accumulation times per point at 0.2 nm intervals using a 1 nm bandwidth. After heating, a further 4°C spectrum was recorded after cooling at a rate of 4°C/min. Buffer baselines were subtracted, data were smoothed, and normalized to mean residue weight ellipticities [θ]_MRW_. Protein concentration was determined from absorption at 280 nm assuming an extinction coefficient of ε_280_ = 50,600 M^−1^cm^−1^. Secondary structure content was estimated using the CDsstr algorithm (Johnson, 1999) as implemented in the Dichroweb server (Whitmore and Wallace, 2004) with the SMP180 reference data set (Lees et al., 2006).

### *In silico* Modeling of the M3-E70A and M3-T272G MHV-68 Mutant Proteins

Models of the M3-E70A and M3-T272G were created using Swiss Model (Waterhouse et al., 2018). To do the modeling, three template structures were selected (PDB IDs: 1MKF, 1ML0, and 2NYZ). Models were built based on a target-template alignment using ProMod3. Coordinates which were conserved between the target and the template were copied from the template to the model. Insertions and deletions were remodeled using a fragment library. The side-chains were then rebuilt. Finally, the geometry of the resulting model was regularized using the GROMOS96 force field (Van Gunsteren et al., 1996). The global and per-residue model quality was assessed using the QMEAN scoring function (Benkert et al., 2011).

### *In silico* Modeling of Complexes of CCL5 and CXCL8 Chemokines With wtM3 MHV-68 Protein and Its E70A and T272G Mutant Forms

Models of the complexes between wtM3 and the CCL5 and CXCL8 chemokines were prepared based on previously determined wtM3–chemokine complex crystal structures. The wtM3–CCL5 complex model was based on the wtM3–CCL2 complex (PDB ID 2NZ1; Alexander-Brett and Fremont, 2007) using a recent structure of CCL5 alone for the chemokine (5COY; Liang et al., 2016). The wtM3–CXCL8 complex model was based on the wtM3–XCL1 complex (2NYZ; Alexander-Brett and Fremont, 2007) using a recent structure of CXCL8 for the chemokine (4XDX, unpublished). To construct each model, the model of a given M3 form or chemokine were superimposed onto the corresponding template protein structure by Cα atom overlap using lsqkab (Kabsch, 1976), part of the CCP4 suite (Winn et al., 2011). For the chemokines, the overlap used 60 Cα atoms for CCL5–CCL2 and 55 for CXCL8–XCL1. These atoms came mainly from the core and C-terminal α-helix of each chemokine structure; all Cα atoms with rmsds over 2.0 Å were excluded from the comparison. Possible clashes were removed by energy minimization against the AMBER99SB-ILDN force field (Lindorff-Larsen et al., 2010) using steepest descent and conjugate gradient minimization as implemented in GROMACS 5.1.3 (Abraham et al., 2015). All hydrogen atoms were removed before the following analyses. The model and crystal structure complexes were analyzed using the COCOMAPS (Vangone et al., 2011) and PISA (Krissinel and Henrick, 2007) servers; interacting residues were also identified using the CCP4 program ncont with a maximum contact distance of 4 Å. The electrostatic surfaces were calculated using APBS (Jurrus et al., 2018) and visualized using PyMOL 1.8.6 and 2.0 (Schrödinger) using the APBS Tools 2.1 plugin.

## Results

### Selection of Amino Acid Residues for Mutagenesis

**Figure 1:**
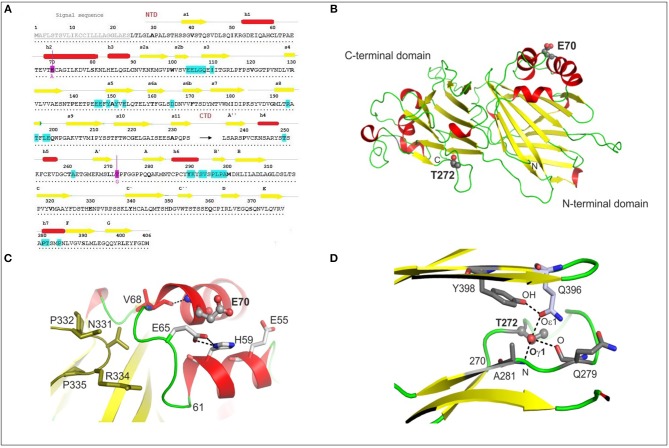
Structural analyses of MHV-68 wtM3 protein. **(A)** The amino-acid sequence of wtM3 including the N-terminal signal sequence, and the N-terminal (NTD) and C-terminal domains (CTD). Residues here and throughout the text are numbered according to the full-length sequence, beginning with the N-terminal signal sequence. Residues thought to be involved in CCL2 binding are in turquoise (Diaz et al., 2009); those chosen for mutation, E70 and T272, are colored magenta. Secondary structure elements, α-helices and β-strands, are marked as red rectangles and yellow arrows, respectively. **(B)** The tertiary structure of the 406-residue MHV-68 M3 (PDB ID 1MKF). Residues E70 and T272, selected for mutation to A and G, respectively, are shown as balls and sticks. **(C)** The conformation of E70 and the surrounding residues E55, E65, H59, and V68 (shown as sticks) in the structure of wtM3. The hydrogen bond between the E70 N and V68 O is shown as a dashed line. Residues 331–335 from the neighboring M3 molecule in the M3 dimer are shown in olive green. **(D)** The hydrogen bond network formed by T272 Oγ1 with Q396 Oε1, A281 N, and Q279 O (all shown as sticks) in the wtM3 structure. The hydrogen bond between Q396 Oε1and the phenolic OH group of Y398 is also shown.

Based on current knowledge of how the M3 structure allows it to bind all chemokine subfamilies, and on the known M3–CCL2 complex crystal structure, we selected two amino-acid residues, E70 and T272, expected to be near the M3 chemokine binding site. E70 is in the M3 NTD (1–234), and T272 is in the CTD (235–406) (Figures 1A,B); both are close to sequences known to be involved in binding chemokine CCL2 (E104–I110, A261, and Y290–A299) (Diaz et al., 2009). According to the known complexes of M3 with CCL2 (PDB ID 2NZ1) and XCL1 (PDB ID 2NYZ) (Alexander-Brett and Fremont, 2007), neither of the selected residues are directly part of the chemokine binding site. In the M3 MHV-68 tertiary structure (PDB ID 1MKF), E70 is a surface-exposed residue and is part of an α-helix containing residues 69–82 (Figure 1B). It is surrounded by the negatively charged residues E55 and E65. E65 is part of a 7-residue loop (61–68) and its side-chain makes a face-to-edge interaction with the imidazole ring of H59 from a neighboring α-helix. The main-chain N of E70 hydrogen bonds to the main-chain O of V68 (Figure 1C). The crystal structure shows that MHV-68 M3 exists as a dimer, and the loop containing residues 61–68 makes contacts with the second M3 molecule in the dimer by hydrogen bonding to the N331 and R334 side-chains. E70 therefore helps in part to stabilize the conformation of both its own and a neighboring α-helix, and also contributes to the conformation of the 61–68 loop contacting the second M3 molecule in the M3 dimeric structure.

Threonine 272 is part of an 11-residue loop (270–280); its Oγ1 side-chain atom is buried within the M3 structure and forms a hydrogen bond network with Q396 Oε1, Q279 O and A281 N (Figure 1D). These interactions help to stabilize not only the conformation of the 11-residue loop, but also, in part, the position of the following two β-strands (residues 281–284 and 396–401).

Before carrying out the mutations, we checked the likely effect of exchanging E for A at residue 70 and T for G at residue 272 on the physico-chemical properties and the surface exposure of the resulting M3 mutant proteins. Using ProtParam (Gasteiger et al., 2005) we found that these mutations should have minimal effect on their pI and instability indices, and that their solubility indices are expected to increase by 8 and 10% for M3-E70A and M3-T272G, respectively (Table S2). Based on the Kyte-Doolittle and Hopp-Woods methods and on surface exposure analysis (Kyte and Doolittle, 1982), a decrease in the hydrophilicity/hydrophobicity profile and the surface exposure area close to the mutation site was calculated for the E70A mutation (Figure S1): this was expected as the E70 side-chain is surface exposed and its replacement by alanine reduces the negative charge on the surface of the protein. Only a minimal decrease in exposed surface area and no change in hydrophobicity/hydrophilicity was calculated for M3-T272G protein (Figure S1).

### Expression and Purification of Recombinant MHV-68 M3 Proteins

**Figure 2:**
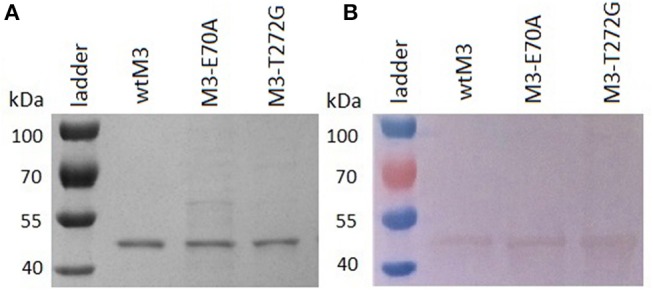
Analyses of purified wtM3 and mutant M3-E70A and M3-T272G proteins. **(A)** SDS-PAGE on a 12.5% gel; **(B)** western blotting.

We transformed *E. coli* Rosetta-gami 2 (DE3) cells with recombinant bacterial expression vectors carrying either wild-type or mutant M3 genes; each of them expressed relatively efficiently in these cells (Figure S2). Analyses of the cell lysate supernatant by SDS-PAGE confirmed that each recombinant M3 was of the expected size, ≈ 44 kDa (Figure 2), with yields of 0.5–2 mg/l culture media. All recombinant proteins, wtM3, M3-E70A, and M3-T272G (Figure 2A), were detected by Western blotting using the primary anti-M3 antibody 1/27 (Figure 2B). All three recombinant M3's were obtained in sufficient quantity for the following studies.

### Binding of Recombinant M3 Proteins to Chemokines

**Figure 3:**
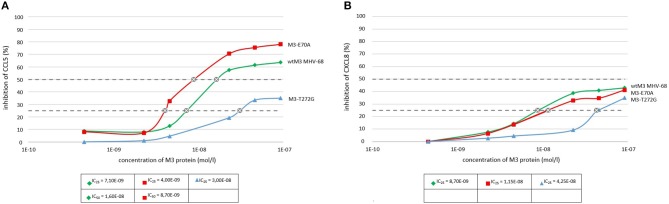
Binding of wtM3 and mutant M3-E70A and M3-T272G proteins to CCL5 **(A)** and CXCL8 **(B)** as determined by ELISA. Each chemokine assay was done in triplicate.

**Figure 4:**
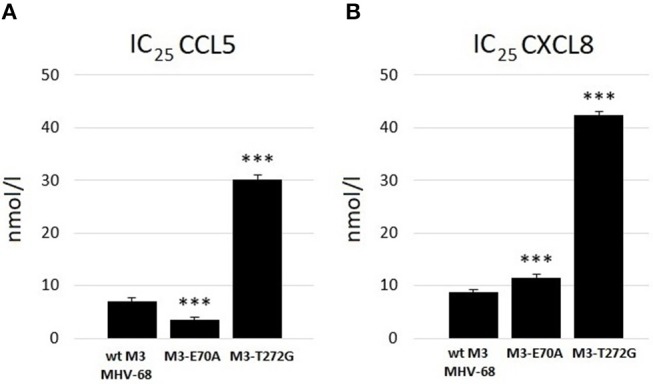
Comparison of the strength of binding of wtM3 and mutant M3-E70A and M3-T272G proteins to **(A)** CCL5 and **(B)** CXCL8. IC_25_ expresses the protein concentration needed to bind 25% of the chemokines used in the assay. The *** above the results of each mutant indicates that the difference from wtM3 is statistically significant at the *p* < 0.001 level. The results shown are an average of three independent experiments.

ELISA assays demonstrated that wtM3 and both mutant forms were able to bind both CCL5 and CXCL8, but with altered affinities. Testing the binding of recombinant M3's with concentration ranges on the order of 10^−10^ to 10^−7^ mol/l to both chemokines we found that wtM3 and M3-E70A, but not M3-T272G, were able to inhibit more than 50% of CCL5 (Figure 3A). The IC_50_ value of M3-E70A and wtM3 was 8.7 nmol/l and 16.2 nmol/l, respectively, indicating that affinity of M3-E70A to CCL5 was roughly twice the affinity of wtM3. As shown in Figure 3B, no of M3 was able to inhibit more than 50% of CXCL8; however, the affinities of wtM3 and the M3-E70A and M3-T272G mutants for both CCL5 and CXCL8 were assessed using IC_25_ values (derived from plots data on Figure 3) allowing the strength of all tested M3-chemokine interactions to be compared. As Figure 4 shows, M3-E70A was able to bind CCL5 with roughly twice the affinity of wtM3 (IC_25_ of 3.5 nmol/l vs. 7.1 nmol/l for wtM3) while CXCL8 showed only a modest reduction of 1.2× (IC_25_ of 11.5 nmol/l vs. 8.7 nmol/l for wtM3). The T272G mutation, however, significantly reduced the affinity of M3 for both chemokines: M3-T272G bound CCL5 with an IC_25_ of 30.2 nmol/l and CXCL8 with 42.5 nmol/l, increases of around 4× and 5×, respectively, over wtM3 (Figure 4). These results suggest that T272 is important for binding to both chemokines.

### *In silico* Models of the wtM3 in Complex With CCL5 and CXCL8

To better understand the ability of M3 to bind the CCL5 and CXCL8 chemokines, and the cause of the diminishing of this binding by the E70A and T272G mutations, we constructed models of complexes between wtM3 and the CCL5 and CXCL8 chemokines.

To date, the only crystal structures of MHV-68 M3–chemokine complexes are with the CCL2 and XCL1 chemokines (Alexander et al., 2002; Alexander-Brett and Fremont, 2007). To find the best template structure for creating our model complexes, we compared the Cα traces of CCL5 and CXCL8 to those of CCL2 and XCL1 (Table 1; Figure S3). All four chemokines had similar overall structural folds, especially in the area of the three-stranded antiparallel β-sheet and the C-terminal α-helix; the largest differences were found in the N-terminal loop and the loop containing residues 30–36 (Figure S3; CCL5 numbering). It is also noteworthy that CXCL8 had the longest C-terminal α-helix. CCL5 and CCL2 had the highest structural similarity, with an rmsd of 0.7 Å. CXCL8 was most similar to XCL1, with an rmsd of 1.4 Å, which was slightly better than its similarity to CCL2 and CCL5, both 1.6 Å. To model the wtM3–CCL5 complex, therefore, we used the wtM3–CCL2 complex as a template and to model the wtM3–CXCL8 complex, we used the wtM3–XCL1 complex.

**Table 1:** Superposition of the Cα traces of the given chemokines on CCL5 and CXCL8.

	**CXCL8 (4XDX)**	**CCL2 (2NZI)**	**XCL1 (2NYZ)**
CCL5 (5COY)	Rmsd: **1.6 Å** Maximum Displacement: **4.4 Å** No. of compared Cα atoms: **41**	Rmsd: **0.7 Å** Maximum Displacement: **2.1 Å** No. of compared Cα atoms: **60**	Rmsd: **0.9 Å** Maximum Displacement: **2.6 Å** No. of compared Cα atoms: **58**
CXCL8 (4XDX)	–	Rmsd: **1.6 Å** Maximum Displacement: **3.4 Å** No. of compared Cα atoms: **56**	Rmsd: **1.4 Å** Maximum Displacement: **3.9 Å** No. of compared Cα atoms: **55**

*L2, XCL1, and CXCL8 Cα traces were overlapped using LSQKAB (Kabsch, 1976)*.

**Figure 5:**
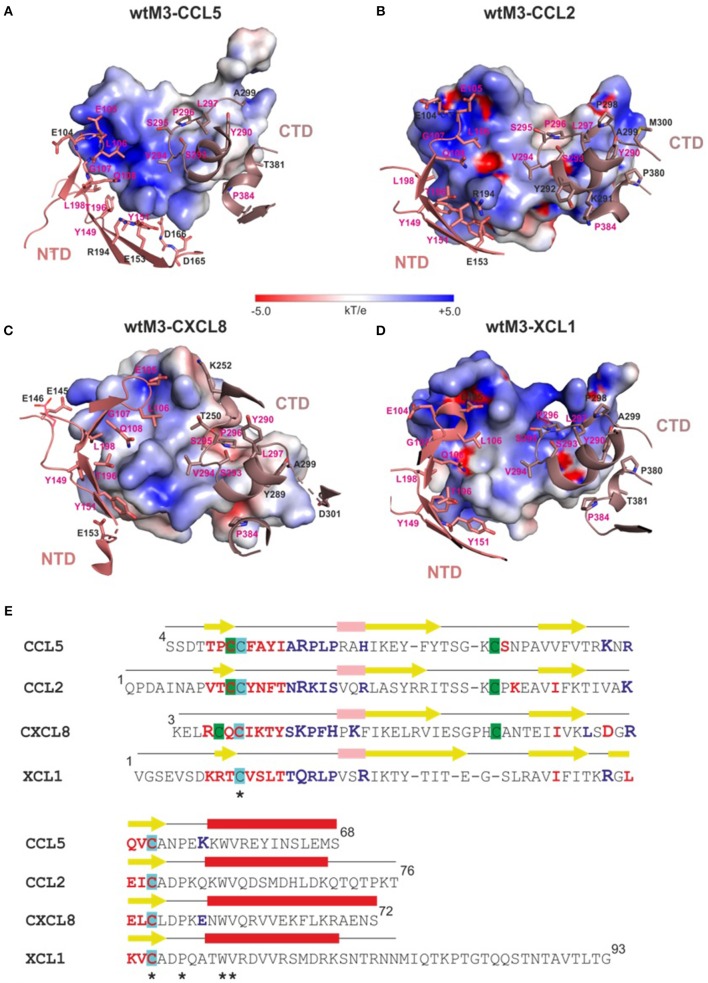
Interfaces of modeled wtM3 complexes with CCL5 and CXCL8 and crystal structures of wtM3 with CCL2 and XCL1. **(A–D)** show those residues of the NTD (salmon) and CTD (gray) of wtM3 within 4 Å of residues from the CCL5 **(A)**, CCL2 **(B)**, CXCL8 **(C)**, and XCL1 **(D)** chemokines. Residues which interact with all four chemokines are labeled in magenta. The electrostatic surface of each chemokine is shown (range ±5 *kT*/e). These surfaces were prepared using APBS (Zhou et al., 2000) and displayed using PyMOL. **(E)** Structure-based sequence alignment of CCL5, CCL2, CXCL8, and XCL1. The secondary structure elements of each chemokine are indicated above its sequence (α-helices are red, 3_10_ helices are pink and β-sheets are yellow). Residues which are identical (*) in all four structures are indicated. Chemokine residues which interact with the M3 NTD and CTD colored blue and red, respectively. Residues which form salt bridges at the M3-chemokine interface are printed larger. Cysteines forming disulphide bonds are undercoated with the same color.

Comparing the modeled wtM3–CCL5 and wtM3–CXCL8 complexes with the experimental wtM3–CCL2 and wtM3–XCL1 complexes revealed differences in the size, amino-acid composition, charge and number of contact residues and salt bridges of the interface areas (Table 2). The wtM3–CXCL8 complex had the largest interface area (1295 Å^2^ corresponding to 4 and 24% of the wtM3 and CXCL8 protein surfaces, respectively), with the largest number of contact residues (25 and 22 for wtM3 and CXCL8, respectively) and the greatest number of hydrogen bonds and charged interactions. Charged interactions are likely to play a crucial role in the formation of this complex, since five salt bridges were identified. Uniquely, of the four analyzed complexes, this was the only one to have charged residues from both the NTD and the CTD of M3 take part (Table 2; Figure 5C); the remaining three complexes had salt bridges to only residues from the NTD.

**Table 2:** Analysis of the M3–chemokine interface areas.

**Complex**	**wtM3–CCL5**	wtM3–CCL2^*^	**wtM3–CXCL8**	**wtM3–XCL1^#^**
^†^Interface area (Å^2^) %wtM3/%chemokine	1,171 (3.6/24.4)	1,042 (3.2/22.5)	1,295.4 (4.1/24)	1,078 (3.4/21.9)
^†^Polar interface area Å^2^ (%)	770.6 (65.8)	653.5 (62.7)	808.1 (62.4)	678.45 (62.9)
^†^Non-polar interface area Å^2^ (%)	400.5 (34.2)	388.6 (37.3)	487.2 (37.6)	400 (37.1)
^†^Contact residues	22/24	25/19	25/22	21/19
^†^Hydrophilic interactions	15	20	19	13
^†^Hydrophobic interactions	12	7	9	10
^*$*^Hydrogen bonds within 3.5 Å	14	16	17	10
^*$*^Charge interactions within 3.5 Å	3 E105/K45 E153/R17 D166/K55	2 E105/K49 E153/R18	6 E105/R47 E145/K20 E153/K15 E194/H18 K252/D45 D301/R6	1 E104/R23

† Performed by the COCOMAPS server (Vangone et al., 2011).

$ Performed by the PISA server (Krissinel and Henrick, 2007).

* ,

# 2NZ1 and 2NYZ PDB structures (side chains A,B, and D) were used.

The wtM3–CCL5 complex had the second largest interface area (1171 Å^2^ corresponding to 3.6 and 24.4% of the wtM3 and CCL5 protein surfaces, respectively). The amino-acid composition of this complex interface is rather different from that of wtM3–CXCL8: it contains more hydrophobic residues and a smaller number of polar and charged residues (Figures 5A,E). This interface has the highest number of hydrophobic interactions and only three salt bridges (Table 2).

The interfaces of the wtM3–CCL2 and wtM3–XCL1 complexes have similar areas (1042 and 1078 Å^2^, respectively; Table 2), and are both smaller than either the wtM3–CCL5 or wtM3–CXCL8 interfaces. Of the four complexes, the wtM3–CCL2 interface has the largest number of hydrophilic interactions, but only two salt bridges (Table 2; Figure 5C), while the wtM3–XCL1 interface has the lowest number of hydrophilic interactions and only one salt bridge (Table 2; Figure 5D).

Common to all four complexes are the charged and polar interactions between Q108, Y149, Y151, T196, S293, and S295 of wtM3 with their unconserved counterparts in each chemokine (Figures 5A–D). There is also a common conserved charged interaction between E105 (for the wtM3–CCL2, wtM3–CCL5, and wtM3–CCXL8 complexes) or E104 (wtM3–XCL1 complex) with a lysine or arginine from the bound chemokine. It is worth noting that most of the negatively charged and polar residues contributed by M3 come from its NTD (L106 is the only non-polar NTD residue) while the CTD contributes some polar residues but also hydrophobic ones. The CTD especially prefers proline, serine, tyrosine, valine and leucine residues. P296 is a good example: in all four wtM3–chemokine complexes, it is buried in a hydrophobic cavity formed by the surface of the interacting chemokine (Figures 5A–D).

It is interesting to note that, aside from one exception in the wtM3–CXCL8 complex (Table 2), M3 always contributes acidic residues (glutamate and aspartate) to complex formation while the chemokines, despite their low amino-acid sequence identity, contribute basic ones (arginine and lysine; Table 2; Figure 5). This is very likely an important part of chemokine recognition by MHV-68 M3 and may be, at least in part, responsible for the “promiscuity” of MHV-68 M3 in chemokine recognition.

### Surface Analysis of the E70A and T272G Mutant Structures

**Figure 6:**
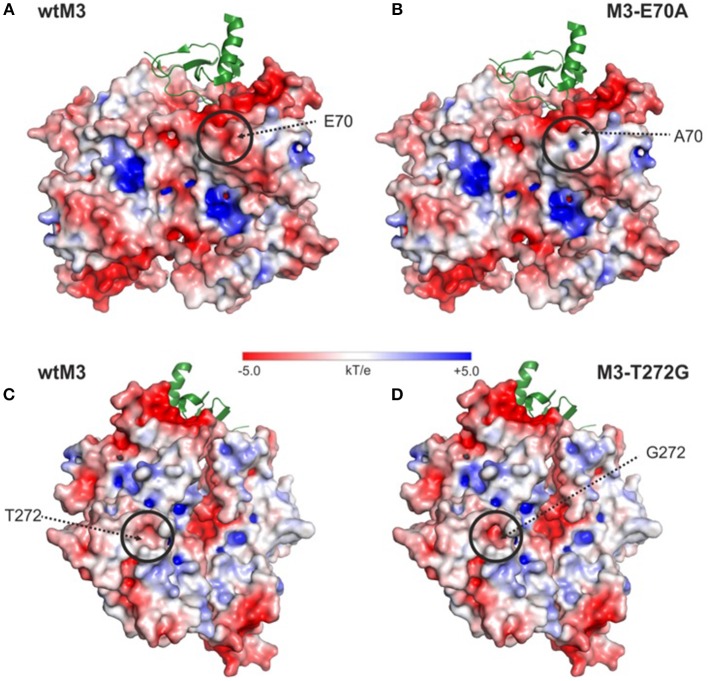
Surface electrostatic charge distribution of wtM3 and modeled M3-E70A and M3-T272G mutants. **(A,C)** Electrostatic charge distribution calculated for the surface of wtM3. **(B,D)** Surface electrostatic charge distribution for the E70A-M3 and T272G-M3 mutants. Areas with a change in charge distribution between the given M3 mutant and the wild-type protein are circled. The interacting chemokine in one of the two chemokine binding sites is shown as a green ribbon. The electrostatic surfaces of all M3's are shown (range ±5 *kT*/e). The surface was prepared using APBS (Zhou et al., 2000) and displayed using PyMOL.

Model structures of the E70A and T272G M3 mutants are almost identical to the structure of wtM3. The main differences are in the amount of exposed surface area and surface charge. This is particularly noticeable in the E70A mutant, where the exposed surface area decreases from 71 Å^2^ to 33.6 Å^2^ and a negative charge in the mutation site and its surrounding environment is eliminated (Figures 6A,B). Surprisingly, the T272G mutation slightly increases the accessible surface area from 5.5 to 8.8 Å^2^ in the mutation site, resulting in a deeper cavity with a higher negative charge (Figures 6C,D). The change in surface charge is due to the exposure of the polar side-chains of Y398 and Q396, which are in contact with T272 in the wtM3 (Figure 1D).

### CD Spectroscopy of wtM3 MHV-68 Protein and Its T272G Mutant Form

**Figure 7:**
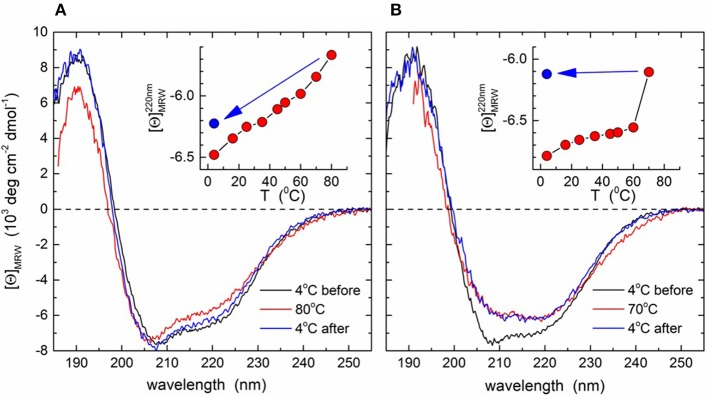
CD spectra of the wtM3 and M3-T272 mutant proteins. Far-UV CD spectra were recorded at increasing temperatures from 4 to 80°C for wtM3 **(A)** or from 4 to 70°C for the M3-T272 **(B)** mutant followed by cooling back to 4°C. Spectra shown are for 4°C before (black line) and after (blue) heating, and at maximum temperature (red). Insets show the ellipticities at 220 nm as a function of temperature; the blue dots indicate [*Θ*]_220_ after cooling.

The wtM3 and mutant M3-T272G recombinant proteins were analyzed by CD spectroscopy. Spectra recorded at 4°C showed a shoulder at 220 nm, a minimum at 208 nm and a maximum at 192 nm (Figure 6). Deconvolution gave an α-helical content of 15–17%, and ~35% of β-strand conformation. These results are in good agreement with the 13 and 38% of α-helix and β-strand content found within the M3 crystal structures (PDB ID 1MKF; Alexander et al., 2002). This indicates that both the wtM3 and T272G adopt a native-like conformation. Spectra recorded at increasing temperatures, up to 80°C, exhibited only very small changes for the wild-type protein, and cooling back to 4°C resulted in a >95% signal recovery (Figure 7A, inset). T272G also produced only small spectral changes upon heating to 60°C; a further spectrum recorded at 70°C, however, showed a significant loss of signal that persisted upon cooling back to 4°C, indicating an irreversible structural change (Figure 7B, inset).

To better understand the role of T272 in thermal stability, we examined its contacts with the rest of the M3 crystal structure. T272 lies in a short, 3-residue loop (residues 271–273) lying between the β-strand-containing residues 268–270 and a short helical turn (Figures 1B,D). Its side-chain is buried and its Oγ1 atom is within hydrogen bonding distance of Q396 Oε1, the A281 main-chain N and the Q279 main-chain oxygen (Figure 1D). Furthermore, Q396 Oε1 hydrogen bonds to the phenolic OH group of Y398. These contacts suggest that the T272 Oγ1 atom forms a hydrogen bond network which stabilizes the surrounding residues (Figure 1D), ultimately making T272 involved in connecting two β-strands (those with residues 268–270 and 396–401), which are part of the two five-stranded β-sheets forming the core of the M3 C-terminal domain. A mutation to glycine would eliminate all these interactions, potentially not only increasing the flexibility of the 271–273 loop, but also the CTD core, leading ultimately to a decrease in M3 thermal stability and possible permanent denaturation, as was observed above 60°C by CD spectroscopy (Figure 7).

## Discussion

The immunomodulatory protein M3 encoded by MHV-68, a pathogen of wild murid species rodents, attracts attention due to its ability to bind promiscuously, but with high affinities, to human and murine chemokines of all chemokine classes. M3 appears to affect the host's immune response to viral infection in at least two different ways: it is able to bind free chemokines, thereby blocking chemokine–receptor interactions by competitive inhibition, and it inhibits the interaction of chemokines with GAGs, thereby affecting the chemotactic gradients needed for directed cell migration (Webb et al., 2004). Due to the complex structure of M3 and the broad spectrum of chemokines that it is able to bind, the functions of individual M3 domains are only poorly understood, but some tentative suggestions have been made. It is thought that the ability of M3 to block the chemokine–GAG interaction lies in its acidic N-terminal domain (*pI* = 4.30 for residues 25 to 230), which electrostatically complements the basic chemokine clusters involved in GAG association. On the other hand, the M3 C-terminal domain engages with residues conserved in different chemokine subclasses which have the same overall spatial arrangement that are involved in receptor binding (Alexander et al., 2002; Webb et al., 2003).

In 2008, Belvončíková et al. found that MHV-72 M3 differed from its MHV-68 counterpart in only a single-amino acid residue located near the CCL2 chemokine-binding area. MHV-72 M3 released into the media from infected BHK-21 cells bound only 11 and 20% of the human CCL5 and CXCL8 chemokines bound by MHV-68 M3. These results suggested that even a single mutation could have an impact on the ability of M3 to bind individual human chemokines. This is consistent with other studies on chemokine binding proteins. Zhou et al. (2000) carried out site-directed mutagenesis to study chemokine receptor CCR5 and found that residues Y10 and K26 play critical structural roles in ligand binding and cell signaling. Petit et al. (2008) prepared a series of mutant CXCR6 proteins and described the effects of individual mutations on receptor function; in particular, the mutations D176N and D274Q made CXCR6 unable to bind to CXCL16. Recently, White et al. (2011) carried out site-directed mutagenesis on 35K-Fc and found that E143 is essential for binding to chemokine CCL5. They also found that an R89A mutation gave 35K-Fc a higher potency for blocking the CCL5 chemokine. Finally, we recently showed that the deletion of the signal peptide (the first 24 residues) in the MHV-68 M3del protein increased M3's binding to CCL5, but not to CXCL8 (Šebová et al., 2017).

Creating *in silico* model complexes of wtM3 with CCL5 and CXCL8 and comparing them to the known X-ray crystal structures of the wtM3–CCL2 and XCL1 complexes (Alexander-Brett and Fremont, 2007) revealed differences in the physico-chemical properties (size, charge, hydrophobicity, and number of interacting residues and charged interactions) of the complex interfaces. Investigating all four interfaces suggested that the ability of MHV-68 M3 to bind a variety of chemokines (Alexander et al., 2002; Webb et al., 2003) arises from a complementary charge distribution between the receptor and ligand, in which M3 contributes mainly negatively-charged residues while the chemokine contributes mainly positively-charged ones. The importance of charged interactions was also shown previously by a kinetic analysis of wtM3 with XCL1 (Alexander-Brett and Fremont, 2007). Our results also showed that there is a principal difference in the interactions between the NTD (mostly charged and polar residues) and the CTD (mostly polar and hydrophobic residues) of M3 with the bound chemokine.

The crystal structure of M3 bound to the CCL2 chemokine revealed several key regions at the interface between M3 and CCL2 (Alexander et al., 2002). Based on the published complex structure of M3 and CCL2, we constructed a series of recombinant mutant proteins targeting residues near the CCL2-binding area and tested their function *in vitro*. To test the chemokine binding properties of these proteins, which specifically bound the anti-M3 1/27 antibody (Šebová et al., 2017), we chose two human chemokines: the inflammatory chemokine CCL5 suggested to be involved in melanoma and in tumor growth and progression (Aldinucci and Colombatti, 2014) and CXCL8, which is highly involved in wound healing and triggering the infiltration of both macrophages and neutrophils in cystic fibrosis (Raman et al., 2007) and is associated with a highly metastatic cancer phenotype (Liu et al., 2016).

Here, based on *in silico* model complexes of wtM3 with CCL5 and CXCL8, we created two M3 mutant proteins with single amino-acid substitutions, E70A and T272G and in *E. coli* cells prepared mutant M3-E70A and M3-T272G proteins using site-directed mutagenesis. Analyzing the ability of each mutant M3 to inhibit both CCL5 and CXCL8 chemokines compared to wtM3 using an ELISA assay showed that neither mutation resulted in a complete loss of M3 function; M3 mutants were still able to bind both chemokines. However, the introduction of each given mutation produced different effects on the affinity of M3 for the tested chemokines. As shown in Figure 3, while the E70A mutation doubled the affinity of M3 for CCL5 relative to wtM3 (IC_50_ of 8.7 vs. 16.2 nmol/l for wtM3; IC_25_ of 3.5 vs. 7.1 nmol/l for wtM3), the T272G mutation reduced M3 affinity for CCL5. M3-T272G was unable to inhibit 50% of CCL5 (Figure 3A) even at the highest tested protein concentration (9x10^−8^ mol/l) used. Thus, only the IC_25_ values of M3-T272G and wtM3 could be compared, showing a significant reduction in M3-T272G affinity for CCL5 (IC_25_ of 30.2 vs. 7.1 nmol/l for wtM3).

Similarly, the inhibition of CXCL8 by all M3's, including wtM3, was lower than that of CCL5, which never reached 50% (Figure 4B). This fact is consistent with the previous finding that M3 displays the lowest affinity for the CXC chemokine subfamily members (Parry et al., 2000; van Berkel et al., 2000; Matúšková et al., 2015). Comparing the IC_25_ values measured in this study allows us to evaluate the affinities of all M3 proteins for both chemokines (Figure 4). Both mutations reduced the affinity of M3 for CXCL8: while E70A showed only a modest reduction of 1.2× (IC_25_ of 11.5 vs. 8.7 nmol/l for wtM3), T272G strongly diminished CXCL8 binding (IC_25_ of 42.5 vs. 8.7 nmol/l for wtM3). To summarize an important result of this study is the discovery that E70 is involved in the interactions of both chemokines, but not substantially, and that the E70A mutation enhanced M3's affinity for CCL5. Further, T272 plays a critical role in both CCL5 and CXCL8 binding and is important for M3 stability, while the T272G mutation causes a significant reduction in binding to both chemokines.

From the structural point of view, E70 is a surface-accessible residue which is sterically close to the chemokine binding site, though it is not part of the interface. Its substitution for an alanine reduces the negative charge on the surrounding area of the NTD (Figure 6B), but only moderately affects binding of CCL5 and CXCL8 (Figure 4). Taken together, we propose that the effect of this mutation on the affinities of CCL5 and CXCL8 very likely does not arise from changes to the M3–chemokine interfaces or from larger changes to the M3 structure, but from changes in the way the two proteins approach and orient each other before full binding actually occurs. Depending on the particular details of the electrostatic surface of the interacting chemokine, therefore, the E70 negative charge might help position the chemokine correctly or slow its correct orientation. Both effects may be present in the current situation. Although CXCL8 does make the most charge-charge interactions in its binding to M3, it actually has a lower overall surface charge than CCL5 (+3 e vs. +5 e). Moreover, in CXCL8 this charge is spread over three different areas of the chemokine, while most of CCL5's charge is concentrated in a single location (in and around the cleft between the C-terminal α-helix and the central β-sheet; cf. Figure 5A vs. Figure 5C). This suggests that for CXCL8 binding, the negative charge on E70 may help to position the chemokine correctly for binding while its absence somewhat slows correct positioning. For CCL5, on the other hand, it may be that E70 slows correct positioning while its absence makes it easier for the positively charged cleft of the chemokine to associate with the negatively-charged NTD, leading to the increase in affinity that we see.

By way of contrast, both chemokines bind substantially less well to the M3-T272G mutant (Figure 4), and in both cases the cause is very likely to be the same. Since T272 is both distant from the binding site and buried, the T272G mutant is likely to exert its effect indirectly. As this mutant has a lower stability than wtM3, we speculate that it might cause some instability in the CTD. Instability in the CTD would be expected to lead to conformational changes to the CTD binding area, thereby weakening the binding of both chemokines equally. In this case, the slightly greater weakening of the CXCL8 interaction might arise from the slightly greater interaction area the CTD makes with this chemokine.

In conclusion, this study provides new insights into the structural basis for M3–chemokine recognition and modulation of M3-chemokine binding. Although not directly part of the M3–chemokine interface, residues E70 and T272 *in vitro* modulate the binding of both human CCL5 and CXCL8 chemokines. The analysis of *in silico* modeled wtM3–CCL5 and wtM3–CXCL8 complexes suggests a different way by which E70 and T272 might affect chemokine binding: E70 *via* negative surface charge reduction and T272 by structural change, resulting in a substantial decrease in chemokine binding as well as a diminishing of M3 stability. The results of this study indirectly indicate that the E70A and T272G mutations might also affect the signaling cascade *in vivo*, however, more experimental work has to be performed to verify this consideration. They show that mutations in M3 modulate (enhance or suppress) the affinity of M3 for a specific chemokine *in vitro*. Our results help better understand the broad specificity of M3 which makes this protein an attractive potential therapeutic in situations where dysregulated immune processes are accompanied by an overabundance of chemokines.

## Data Availability

This manuscript contains previously unpublished data. The name of the repository and accession number are not available.

## Author Contributions

RŠ, MK, VB-H, JB, KB, and IN performed experiments, and analyzed and interpreted the data. MK, VB-H, and KB wrote the manuscript. MK and VB-H conceived and directed the study. All authors contributed to discussions.

### Conflict of Interest Statement

The authors declare that the research was conducted in the absence of any commercial or financial relationships that could be construed as a potential conflict of interest.

## Supplementary Material

Figure S1 Hydrophobicity/hydrophilicity profile in the vicinity of the mutation sites. The mutation of M3 residues E70 to A70 and T272 to G272 resulted in changes to the predicted hydrophobicity **(A)** and hydrophilicity **(B)** of the protein in the area surrounding the mutations. A reduction in hydrophilicity was associated with the E70 mutation (indicated by blue).

Figure S2 SDS-PAGE (12.5%) analysis of the expression in *E. coli* Rosetta-gami 2 (DE3) cells and purification by IMAC of wtM3 and its mutants. **(A)** wtM3; **(B)** M3-E70A; **(C)** M3-T272G. E indicates fractions eluted from the affinity column, the arrow indicates wtM3 size (44 kDa).

Figure S3 Superposition of the Cα traces of the CCL5 (blue), CCL2 (green), CXCL8 (red) and XCL1 (orange chemokines). The greatest differences are seen in the N-terminus and in the loop containing residues 30–36 (CCL5 numbering). Details of the superposition are given in Table 1. Cysteines forming disulfide bonds are indicated as balls and sticks and numbered; Sγ atoms are colored in yellow, Cα and Cβ are colored according to the chemokine.

Table S1 Forward and reverse primers used to prepare the M3 mutant constructs.

Table S2 Predicted properties of each M3 protein.

## Abbreviations

BHK-21: baby hamster kidney cells
CCL2/MCP-1: chemokine ligand 2/monocyte chemoattractant protein 1
CCL5/RANTES, chemokine ligand 5/regulated upon activation: normal T cell expressed and secreted
CD spectroscopy: circular dichroism spectroscopy
CXCL8/IL-8: chemokine ligand 8/interleukin-8
ELISA: enzyme-linked immunosorbent assay
GAG: glycosaminoglycans
IMAC: immobilized metal affinity chromatography
IPTG: isopropyl β-d-1-thiogalactopyranoside
M3: M3 protein
MHV-68: murine gammaherpesvirus 68
MRW: mean residue weight
NTD or CTD: N-terminal or C-terminal domain of M3 protein
vCKBP: viral chemokine-binding protein
wtM3: wild-type M3 protein.
