# Supplementary figures and images for: Residue Mutations in Murine Herpesvirus 68 Immunomodulatory Protein M3 Reveal Specific Modulation of Chemokine Binding
Source: Front Cell Infect Microbiol. 2019 Jun 25;9:210. doi: 10.3389/fcimb.2019.00210 (PMC6603146; doi:10.3389/fcimb.2019.00210)

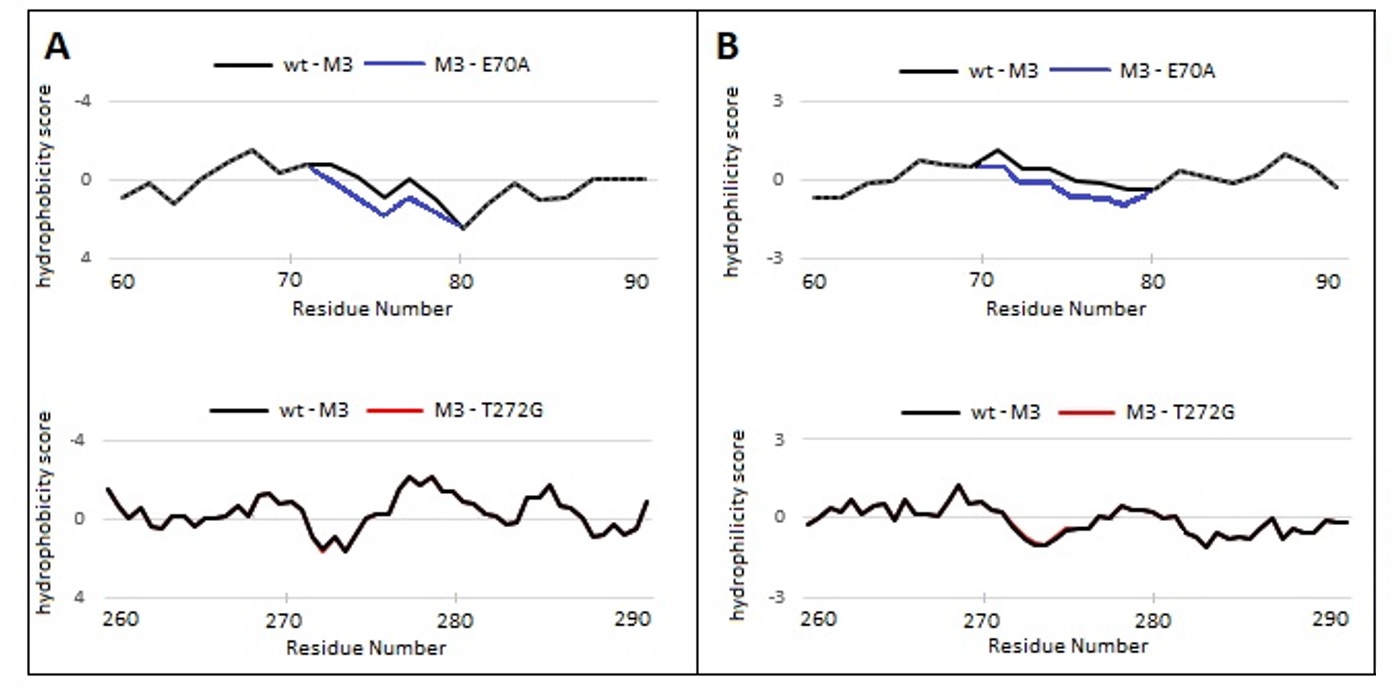

Supplement: Figure S1 — Hydrophobicity/hydrophilicity profile in the vicinity of the mutation sites. The mutation of M3 residues E70 to A70 and T272 to G272 resulted in changes to the predicted hydrophobicity (A) and hydrophilicity (B) of the protein in the area surrounding the mutations. A reduction in hydrophilicity was associated with the E70 mutation (indicated by blue). [file Image_1.JPEG]

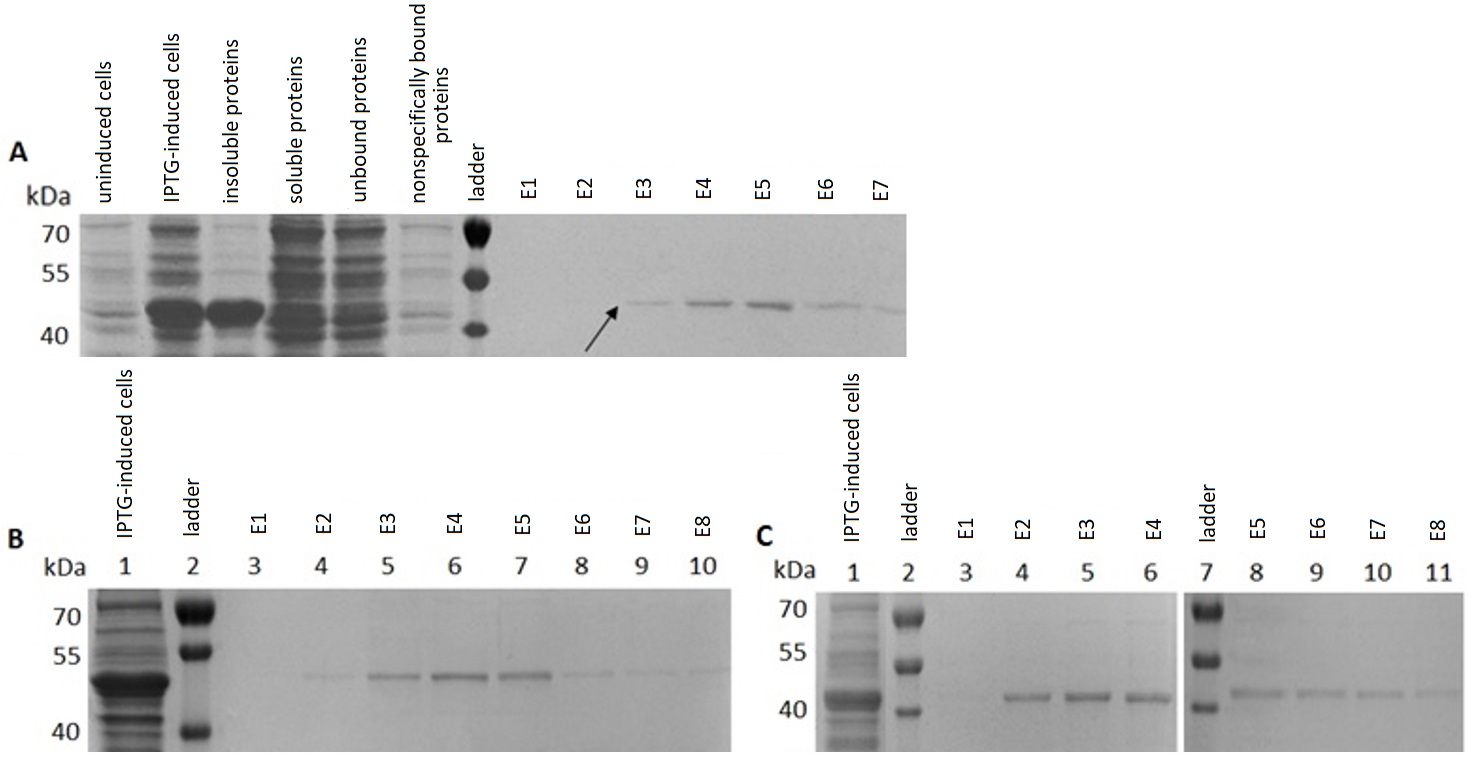

Supplement: Figure S2 — SDS-PAGE (12.5%) analysis of the expression in E. coli Rosetta-gami 2 (DE3) cells and purification by IMAC of wtM3 and its mutants. (A) wtM3; (B) M3-E70A; (C) M3-T272G. E indicates fractions eluted from the affinity column, the arrow indicates wtM3 size (44 kDa). [file Image_2.JPEG]

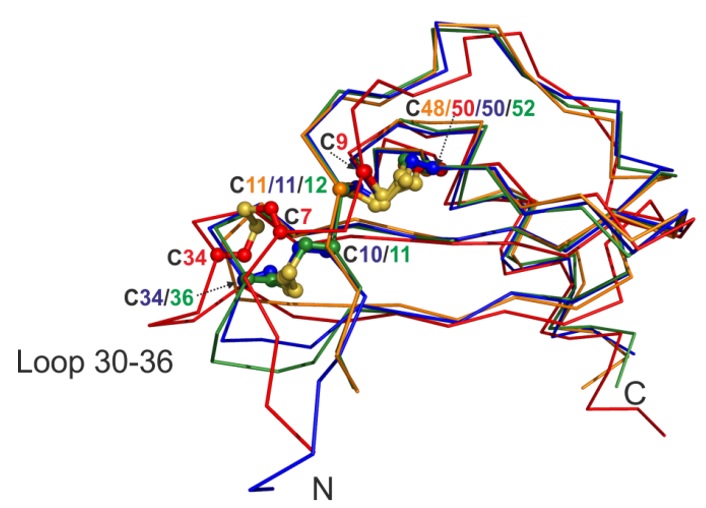

Supplement: Figure S3 — Superposition of the Cα traces of the CCL5 (blue), CCL2 (green), CXCL8 (red) and XCL1 (orange chemokines). The greatest differences are seen in the N-terminus and in the loop containing residues 30–36 (CCL5 numbering). Details of the superposition are given in Table 1. Cysteines forming disulfide bonds are indicated as balls and sticks and numbered; Sγ atoms are colored in yellow, Cα and Cβ are colored according to the chemokine. [file Image_3.JPEG]

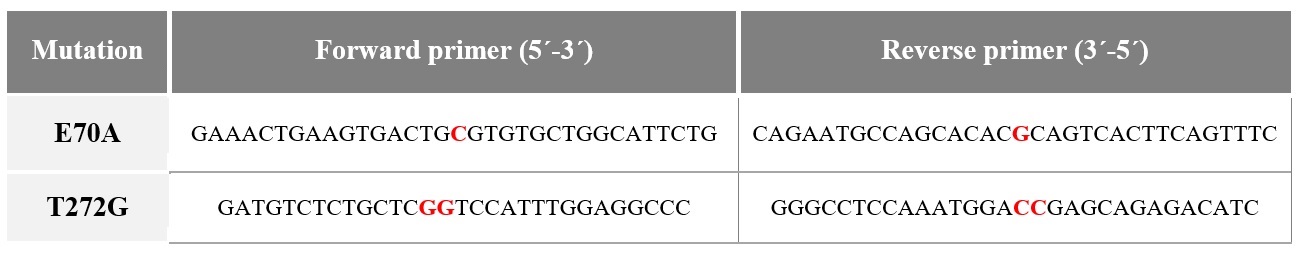

Supplement: Table S1 — Forward and reverse primers used to prepare the M3 mutant constructs. [file Image_4.JPEG]

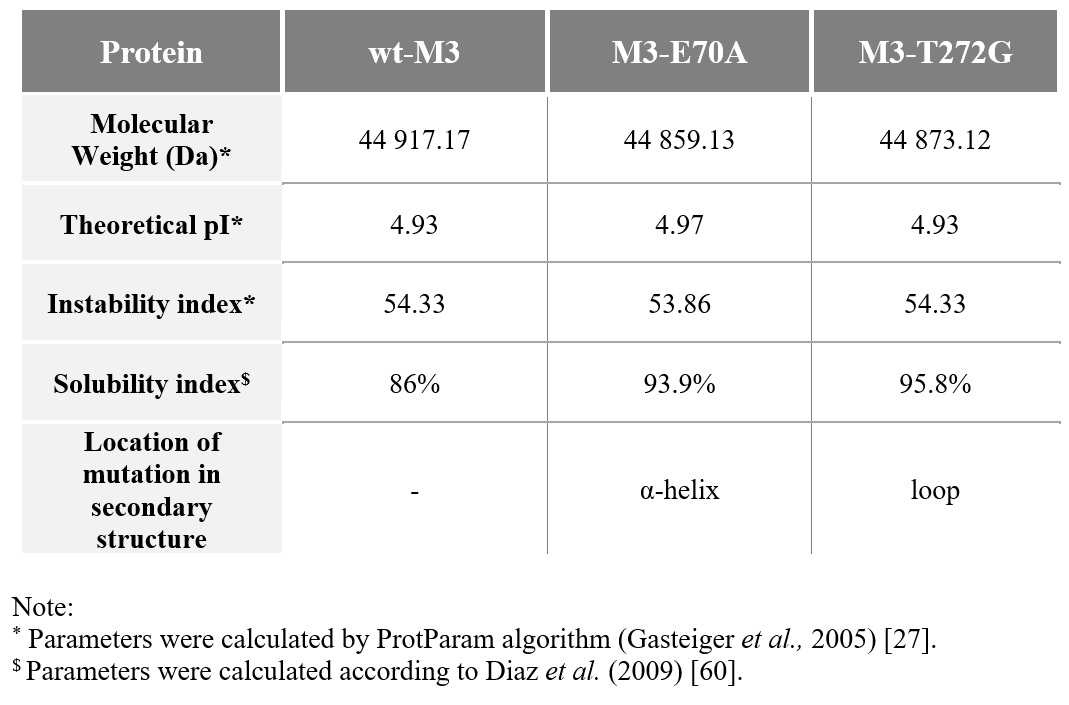

Supplement: Table S2 — Predicted properties of each M3 protein. [file Image_5.JPEG]
